# Experimental and RSM-Based Process-Parameters Optimisation for Turning Operation of EN36B Steel

**DOI:** 10.3390/ma16010339

**Published:** 2022-12-29

**Authors:** Ramesh Kumar, Ashwani Kumar, Laxmi Kant, Arbind Prasad, Sandeep Bhoi, Chandan Swaroop Meena, Varun Pratap Singh, Aritra Ghosh

**Affiliations:** 1 Department of Mechanical Engineering, Saharsa College of Engineering, Saharsa 852201, Bihar, India; 2 Technical Education Department Uttar Pradesh Kanpur, Kanpur 208024, Uttar Pradesh, India; 3 Department of Mechanical Engineering, Government Engineering College Bhojpur, Patna 800024, Bihar, India; 4 Department of Mechanical Engineering, Katihar Engineering College (under Department of Science & Technology, Government of Bihar), Katihar 854109, Bihar, India; 5 Department of Mechanical Engineering, Parala Maharaja Engineering College, Berhampur 761003, Odisha, India; 6 CSIR-Central Building Research Institute, Roorkee 247667, Uttarakhand, India; 7 Academy of Scientific and Innovative Research (AcSIR), Ghaziabad 201002, Uttar Pradesh, India; 8 Department of Mechanical Engineering, School of Engineering, University of Petroleum and Energy Studies, Dehradun 248007, Uttarakhand, India; 9 Faculty of Environment, Science and Economy (ESE), Renewable Energy, Electric and Electronic Engineering, University of Exeter, Penryn, Cornwall TR10 9FE, UK

**Keywords:** cutting-tool wear, process parameters, turning operation, optimisation, response-surface method, EN36B steel

## Abstract

The main objective of this article is to perform the turning operation on an EN36B steel work-billet with a tungsten carbide tool, to study the optimal cutting parameters and carry out an analysis of flank-wear. Experimental and simulation-based research methodology was opted in this study. Experimental results were obtained from the lab setup, and optimisation of parameters was performed using RSM (response surface methodology). Using RSM, cutting-tool flank-wear was optimised, and the cutting parameters which affect the flank wear were determined. In results main effect plot, contour plot, the surface plot for flank-wear and forces (Fx, Fy and Fz) were successfully obtained. It was concluded that tool flank-wear is affected by depth of cut, and that flank-wear generally increases linearly with increasing cutting-speed, depth of cut and feed-rate. To validate the obtained results, predicated and measured values were plotted and were in very close agreement, having an accuracy level of 96.33% to 98.92%.

## 1. Introduction

In metal cutting, tribology plays an important role, as it decides the surface quality as well as the performance of the product during operation. Wear, friction and surface roughness constitute an integral part of tribology [1,2,3]. Four basic elements, i.e., body, counter-body, ambient medium, and interface material make up the structure of the tribological system. The machining of the materials is an example of the open-type tribological system in which the body can be replaced by a tool, the counter-body can be replaced by work material, and the interface material can be replaced by the lubricant used for cooling purposes in the machining. One of the most commonly used techniques for the fabrication of components is machining; during the machining of components, excessive wear of the cutting tool gives rise to distortion in the dimensions of manufactured components, and increases the level of scrapped material [4,5]. Hence, it becomes necessary to monitor the cutting-tool wear. Turning is an example of the machining process, and in this process, the extra materials on the workpiece can be removed by a single-point cutting tool [6]. The main reason for cutting-tool failure is a mechanical failure of the cutting tool, which may be due to excessive forces and shocks. The nature of this type of failure of the cutting tool is catastrophic, and hence this type of failure is detrimental. It occurs due to the intensive temperature and the stresses, which are quick to cause dulling, due to plastic deformation [7,8,9]. This type of failure is unwanted and detrimental, and takes place at a very fast rate. Another type of failure that occurs in the tool is gradual wear of the cutting tools, which causes failure of the flank surface and the rake surface of the tool [10,11,12].

The two modes of tool failure, i.e., the mechanical failure of the tool and the failure of the tool due to intensive stresses and temperature, are very harmful to the tool as well as to the job. Therefore, these types of tool failures need to be stopped by taking some necessary action, such as by using suitable tool geometry and tool materials. Furthermore, tool failure due to gradual wear is inevitable and cannot be stopped [13,14,15]. Gradual wear of the tool can only be slowed down, to increase the life of the tool. In the machining process, the cutting tool needs to be replaced just before the tool is going to fail or just after the tool has failed. We need criteria to judge or to decide whether a tool is about to fail or has failed [16,17,18]. Researchers have performed the optimisation study of cutting tools by turning for surface roughness. They have determined tool wear using force signals, as we can extract more information about the machining process from the force signals [19,20,21,22,23]. In recent studies, authors have studied the process parameters using RSM, which has been adopted for the current study. In the present research work, the relationship among the feed force, cutting-force components (the ratio of the force components, F_X_ and F_Z_) flank-wear, and other cutting parameters (cutting speed, feed, depth of cut and diameter) was established by performing a series of experiments for accessing tool characteristics [24,25,26,27]. Tool-life performance, the wear mechanism, and surface roughness were also studied by Motorcu et. al., by using input parameters. They investigated the wear behaviour of a ceramic tool Al_2_O_3_, and, Al_2_O_3_ coated with TiN and CBN/TiC for the machining of the hardened steel AISI 52100, and surface roughness was compared under various conditions. Joardar et al. made an attempt to find the effect of specific cutting variables in straight-turning, on cutting forces. The materials used in the straight-turning operation were the metal matrix composites of aluminium under dry cutting conditions. Some of the influencing parameters selected are as follows: depth of cut, cutting speed, and weight percentage of SiCP [28,29,30,31].

Sikdar and Chen successfully established a relationship between the above components. They further conclude that adhesive and abrasive actions between the workpiece and the tool, cause the flank wear. First, there the flank wear starts at the tip of the cutting tool, and then it increases widthways, thus resulting in a wear land. Sun et. al. and Oraby and Hayhurst studied a model for finding tool life and wear in terms of the variation of a ratio of the force components acting at the tooltip, which were established by nonlinear regression analysis. In continuation, Noordin et. al., using RSM, studied the performance of carbide tools [32,33,34,35]. A comprehensive model that relates the wear to the change in the cutting force was proposed and tested. They describe the performances of a multilayer, tungsten carbide tool using response-surface methodology. The cutting test was performed with a constant depth of cut under dry conditions [36,37,38,39,40]. Yang and Tang considered a practical turning case, which could be used for tool-wear estimation when the crater-wear effect is negligible (or be combined with the crater-wear model when the crater-wear effect is influential). They showed successfully that the method which provides an efficient and systematic methodology in the optimum designing of cutting parameters is the Taguchi method. Kumar et al. studied the optimization techniques for different engineering problems. Bhoi et al. successfully carried out condition monitoring and a delamination study on dynamic analysis. They investigated the wear failure [41,42,43,44].

In the literature, authors have studied cutting-tool failures due to wear. In continuation, this article presents a tool-wear study using experimental and simulation results [45,46,47,48]. In this article, a turning operation was performed on an EN36B steel work-billet with a tungsten carbide tool and process parameters such as the feed components of force (F_X_, F_Y_ and F_Z_), cutting speed, feed rate and depth of cut. These were studied experimentally and results were validated using the RSM tool.

## 2. Materials and Methodology

In the current study, we adopted the design of experiments (DOE) to plan and optimise the experiments required. A test or series of tests are called upon to solve any problem with the help of experiments, and this plays a major role in the DOE. For designing the factorial experiments, experimental trials needed to be performed with all combinations of the levels of factors. However, with the help of the DOE, there is the capability to simultaneously investigate the effects of different variables on the response, i.e., on the output variable. The experiments in the DOE consist of many tests in which input variables are altered accordingly, and the data are collected after the tests. Tool wear affects the feed components of the force (F_X_) and the radial components of force ($F_{y}$). The frictional and sliding condition between the workpiece and the tool is closely related to the components of force F_X_ and F_Y_. The respective friction condition of F_X_ and F_Y_, and their combined effects, can be measured by F_Z_. The vertical component of force, F_Z_ can be measured in terms of the power and torque required for the operation of the lathe.

In the initial stage, the value of the component of forces, i.e., F_X_ and F_Z_ were almost the same. After the progress of the machining operation, and due to the wear of the cutting-edge of the tool, the values of the components of the forces no longer remain the same. The values of the forces, i.e., F_X_ and F_Z_ depends on the condition of distribution of the wear-scars on the cutting-edge of the tool. Generally, on the tool flank-face, the wear scars are not distributed evenly. The nose wear (NW) of the tool is mainly responsible for the change in the value of the radial component of force, i.e., F_Y_. The presence of wear on the nose and flank of the tool affects the force component, i.e., F_X_. The force components associated with a particular area are mostly influenced by the domination of wear in that area. At the point where the tool has maximum projection into the workpiece, the nose-wear was measured. The flank-wear was measured at the maximum wear land on the tool flank. According to ISO, the average value of wear at failure VB (width of wear land) = 0.30 mm. The maximum value of VB is 1.7 mm, which is permissible in flank-wear (VB). Many professional uses the DOE to find the product components and process conditions that affect quality. After this, they try to find the input factors or variables that need to be regulated for obtaining the optimum point of the desired objective. The level that was chosen for each cutting parameter is given in Table 1. The parameter levels were selected as recommended by the tool manufacturer. Cutting parameters and their levels are given in Table 1. Three cutting parameters at three levels led to 27 tests. The plan of the experiments as designed using concepts of DOE is given in Table 2. A full factorial design using Minitab15 software (Academic version, 2020, India) was created, and the experiments were performed using this plan. Cutting speed, feed rate and depth of cut are the three different levels ($3^{3}=27$) for which experiments were performed. At the last stage the predicated values and measured values were compared, and the graph was plotted for the results analysis.

**Figure 1:**
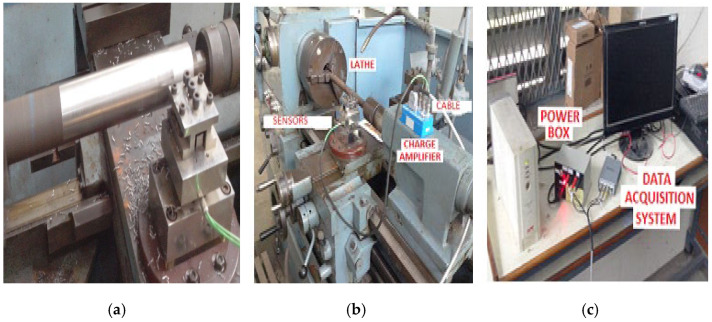
Experiment setup for wear analysis of tungsten carbide tool: (**a**) tool post, (**b**) different parts on the lathe, and (**c**) setup of the data-acquisition system.

The experiments were performed using the single-point cutting tool, and the experimental setup on the lathe with the single-point cutting tool is shown in Figure 1. Figure 1a shows the tool-post of the lathe, and the complete setup of the lathe machine is shown in Figure 1b. The data-acquisition setup used for measuring the tool wear is shown in Figure 1c.

In the current study, EN36B steel of dimension 50 mm × 400 mm was used as a workpiece. The purpose of selecting EN36B as a workpiece material is because of its abundance of use in manufacturing. It is generally used for manufacturing automobile components such as worm shafts, gears, spline shafts, and pinion shafts. It is also used in the manufacturing of heavy-vehicle transmission and auto components, aircraft gear mining, chuck jaws, steering worms, pinions, cogs, and gudgeon pins. The tool holder SCLCR1212F09 is used with a carbide tool insert, CCMT09T302. After performing every experiment, the insert was changed, to minimise the effect of tool wear on the material removal rate and cutting forces. A piezoelectric dynamometer (KISTLER Maker, Kistler Instruments India Pvt. Ltd., Chennai, India) was used for measuring the cutting forces.

The weight of the workpiece is precisely measured with an accuracy of up to a milligram, with the help of a precision weighing machine (MT India Pvt. Ltd., Mumbai, India). Tool wear (depth of flank-wear VB) is measured with the help of a metallurgical microscope (Delhi Metco, New Delhi, India). The chemical composition of the EN36B used for the machining process is listed in Table 2. The EN36B core strength can be up to 1230 N/mm^2^, and the hardness value is 255 (Brinell hardness RC26).

## 3. Results and Discussion

The effect of the different design parameters on the quality of the machining process can be analysed by using the analysis of variance (ANOVA). For flank-wear, the results of the analysis of variance obtained at the 5% level of significance were analysed. The magnitude and existence of some of the physical quantities could not be quantified directly, but by using the ANOVA it was possible to find them. The ANOVA is not only able to quantify physical quantity, but can also analyse its effect. An example of this might be a force, which is neither is held nor seen. However, it can not only be detected, but also quantified, with the help of these effects, such as deformation, deflection, strain, pressure, etc., which can be quantified. The signals of these effects need to be conditioned properly, prior to analysis, for accurate, easy measurement of these effects. The values of the speed, feed and depth of cut obtained, are given in the form of an observation table in Table 3. The result of analysis of variance (ANOVA) for VB, F_X_, F_Y_ and F_Z_ is tabulated in Table 4.

The purpose of the analysis of variance (ANOVA) is to find which design parameters significantly affect quality characteristic. The results of the analysis of variance (ANOVA) at a 5% level of significance for flank-wear, F_X_, F_Y_ and F_Z_ are shown in Table 4.

### 3.1. Main Effects Plot for VB, F_X_, F_Y_ and F_Z_

**Figure 2:**
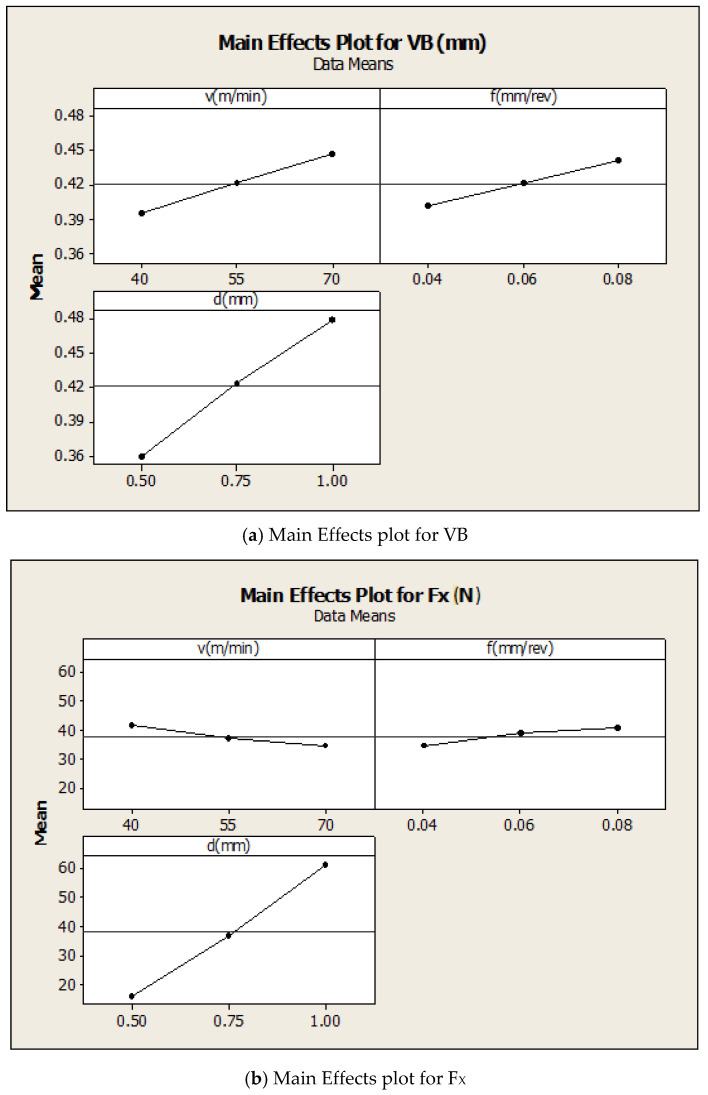

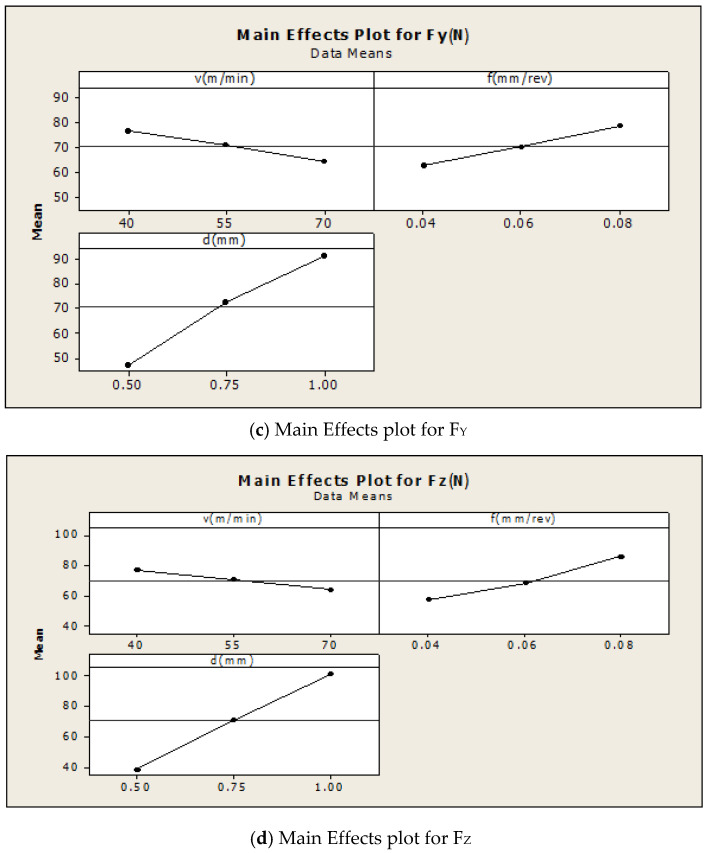
Main Effects Plot for VB, F_X_, F_Y_ and F_Z_.

The main effects plot for VB is given in Figure 2a. From the figure, it is found that flank-wears i.e., wear of the flank, generally increases linearly with an increase in cutting speed, depth of cut and feed rate. The main effects plot for F_X_ is given in Figure 2b. From this figure, it is found that axial force generally decreases by increasing the cutting speed, but increases by increasing the feed rate and depth of cut. The main effects plot for F_X_ is given in Figure 2c. From this figure, it is found that radial force generally decreases by increasing the cutting speed, but increases by increasing the feed rate and depth of cut. The main effects plot for F_Y_ is given in Figure 2d. From this figure, it is found that the tangential cutting force generally decreases by increasing the cutting speed, but increases by increasing the feed rate and depth of cut.

### 3.2. Contour Plot for VB, F_X_, F_Y_ and F_Z_

**Figure 3:**
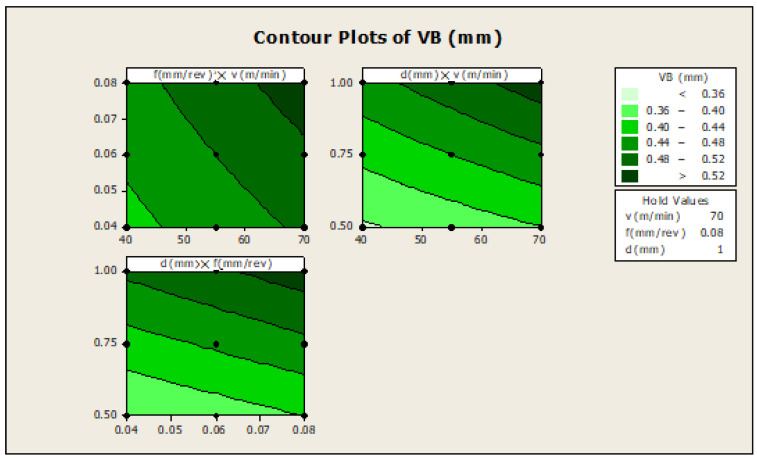
Contour plots for VB.

The pair of input variables with the same response value can be represented by a pair of straight lines or contour lines. The contour plot of the VB is shown in Figure 3: $\left(f\times v\right)$, shows how the variables feed rate and cutting speed, are related to the VB. The constant value, i.e., the high-value one, was used for the depths of cut, (d). In the upper right-hand corner of the graph, i.e., in the darkest region, the highest value of the response is 0.52.

Figure 3: $\left(d\times v\right)$, the plot shows how the variables depth of cut and cutting speed, are related to the VB, while other factors such as feed rate are held constant, at a highest value of 0.08. In the upper right-hand corner of the graph, i.e., in the darkest region, the highest value of the response is greater than 0.52. In $\left(d\times f\right)$, the plot gives information of how variables such as depth of cut and feed rate are related to the VB, while other factors such as cutting speed are held constant, at a high value of 70.

**Figure 4:**
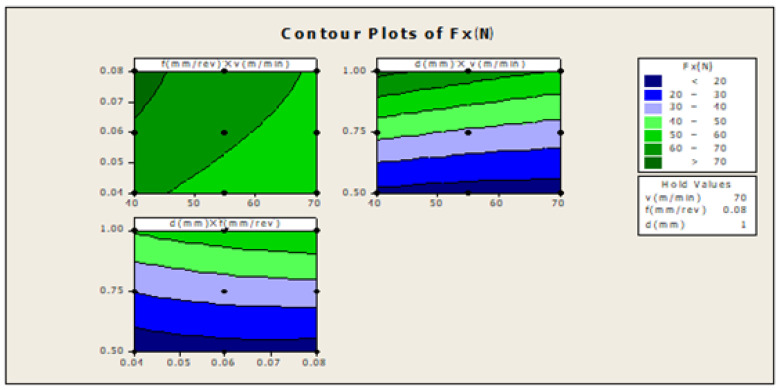
Contour plot for Fx.

In Figure 4 $\left(f\times v\right)$, the plot indicates how variables such as feed rate and cutting speed are related to the F_X_, while other factors such as depth of cut (d) are held constant, at a high value of 1. The response is at its highest (greater than 70) in the darkest region of the graph (the upper left-hand corner). In $\left(d\times v\right)$, the plot indicates how variables such as depth of cut and cutting speed are related to the F_X_, while others such as the feed-rate factor are held constant, at a high value of 0.08. The response is at its highest (greater than 70) in the darkest region of the graph (the upper left-hand corner). In $\left(d\times f\right)$, the plot indicates how variables, such as depth of cut and feed rate are related to the Fx while other factors such as cutting speed are held constant, at a high value of 70. The response is at its highest (between 50 and 60) in the dark region of the graph (the uppermost corner).

**Figure 5:**
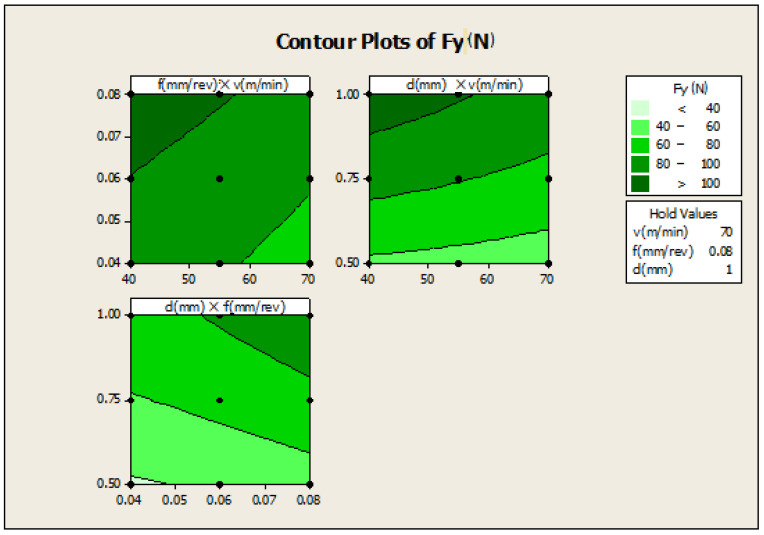
Contour plot for Fy.

Figure 5: (f×v)—the plot indicates how variables such as feed rate and cutting speed are related to the Fy, while other factors such as depth of cut (d) are held constant, at a high value of 1. The response is at its highest (greater than 100) in the darkest region of the graph (the upper left-hand corner); $\left(d\times v\right)$—this plot indicates how variables such as depth of cut and cutting speed are related to the Fy, while other factors such as feed rate are held constant, at a high value of 0.08. The response is at its highest (greater than 100) in the darkest region of the graph (the upper left-hand corner); $\left(d\times f\right)$—this plot indicates how variables such as depth of cut and feed rate are related to the Fy, while other factors such as cutting speed are held constant, at a high value 70. The response is at its highest (greater than 100) in the dark region of the graph (the upper right-hand corner).

**Figure 6:**
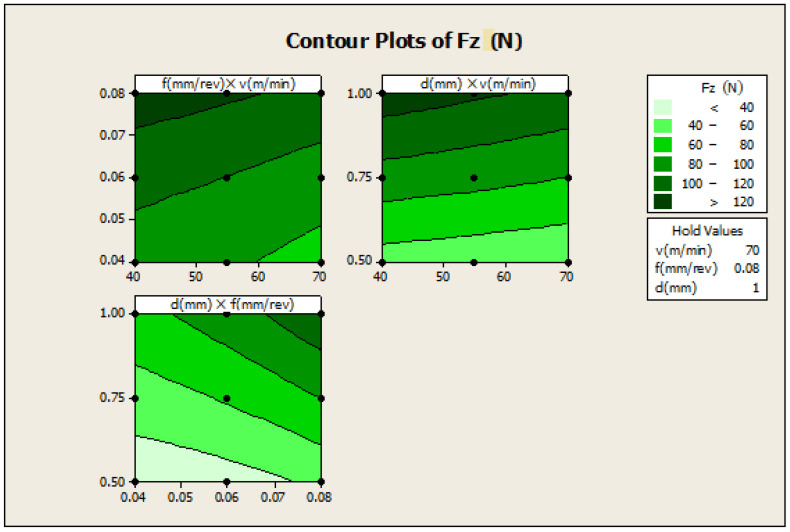
Contour plot for Fz.

In Figure 6, $\left(f\times v\right)$ the plot indicates how variables such as feed rate and cutting speed are related to the Fz, while other factors such as depth of cut (d) are held constant, at a high value of 1. The response is at its highest (greater than 120) in the darkest region of the graph (the upper left-hand corner); $\left(d\times v\right)$—this plot indicates how variables such as depth of cut and cutting speed are related to the F_Z_, while other factors such as feed rate are held constant, at a high value 0.08. The response is at its highest (greater than 120) in the darkest region of the graph (the upper left -hand corner); $\left(d\times f\right)$—this plot indicates how variables, depth of cut and feed rate are related to the F_Z_, while other factors such as cutting speed are held constant at a high value of 70. The response is at its highest (greater than 120) in the dark region of the graph (the upper right-hand corner).

### 3.3. Surface Plot for VB

**Figure 7:**
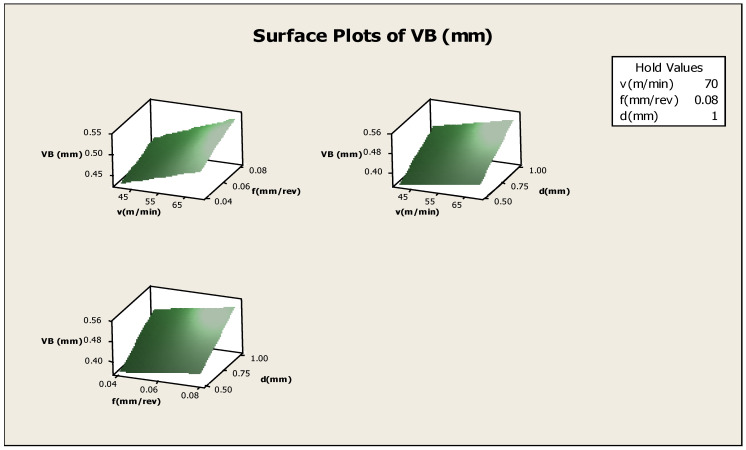
Surface plots for flank-wear.

**Figure 8:**
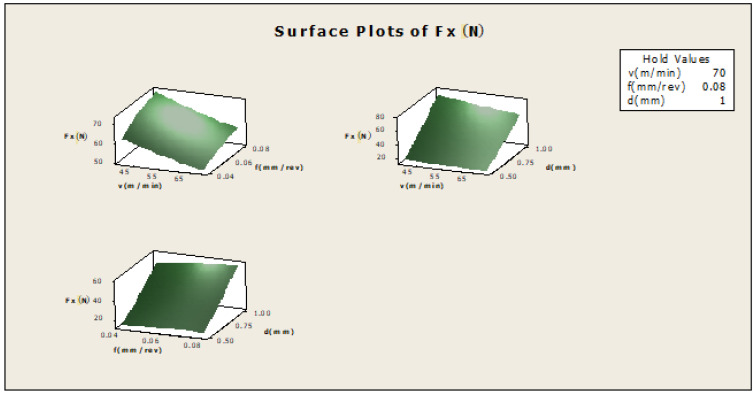
Surface plot for Fx.

**Figure 9:**
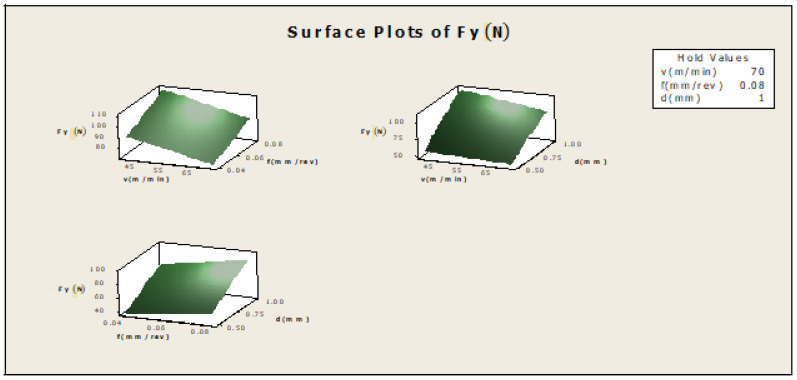
Surface plot for F_Y_.

**Figure 10:**
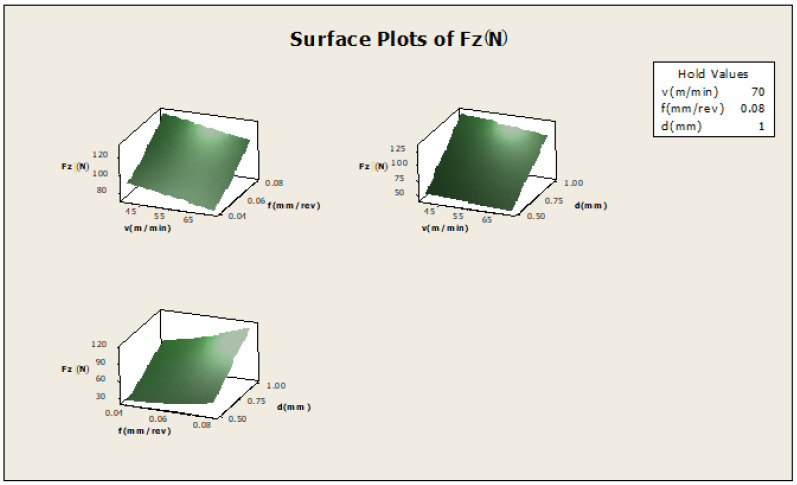
Surface plot for F_Z_.

The aim of this work is to understand the variation in the response in a particular direction, by changing the design variables. Generally, the response surface is a graphical representation, and with the help of a graph, the response can be visualised. The shape of the response surface, i.e., the ridgelines, valleys, and hills, can be visualised with the help of the graph. One can draw a three-dimensional plot for better understanding, and the side view shows the response surface. This type of plot is known as the response-surface plot. Hence, a clear snap of the response surface can be obtained with the help of a three-dimensional surface plot. The surface plot for VB is shown in Figure 7, Figure 8, Figure 9 and Figure 10.

### 3.4. Modelling of VB, F_X_, F_Y_ and F_Z_

The coefficient of correlation (R^2^) was used to estimate the relevance and validity of the obtained results. An R^2^ value shows how well the estimated model fits the data. The range of the values of R^2^ is 0 ≤ R^2^ ≤ 1. For better fitting of the experimental data with the regression equation, the value of R^2^ should be near 1. We used a linear regression analysis to fit the data. The VB model is given by the mathematical expression, and it can be expressed by Equation (1):

(1) $$VB=0.0904+0.00171\;v\;\left(m/min\right)+0.969\;f\;\left(mm/rev\right)+0.237\;d\;\left(mm\right)$$

Its coefficient of correlation, R^2^ (adj.) is 96.33%. The main aim is to check whether this model is accurate or not. To do this, separate experiments were performed and their results were compared for predicted and measured values. We found that the predicted value of VB is almost nearer to the measured value. Hence, we can say that Equation (1) represents the data with 96.33% accuracy.

The F_X_ model is given by:

(2) Fx=−49.48+0.441v+6.889f+113.84d−0.757v×d

**Figure 11:**
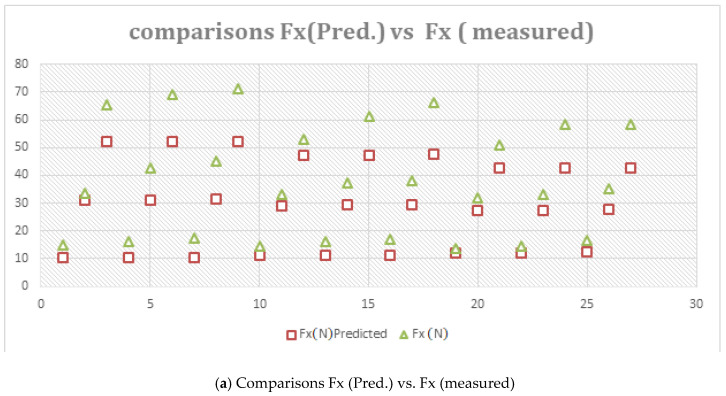

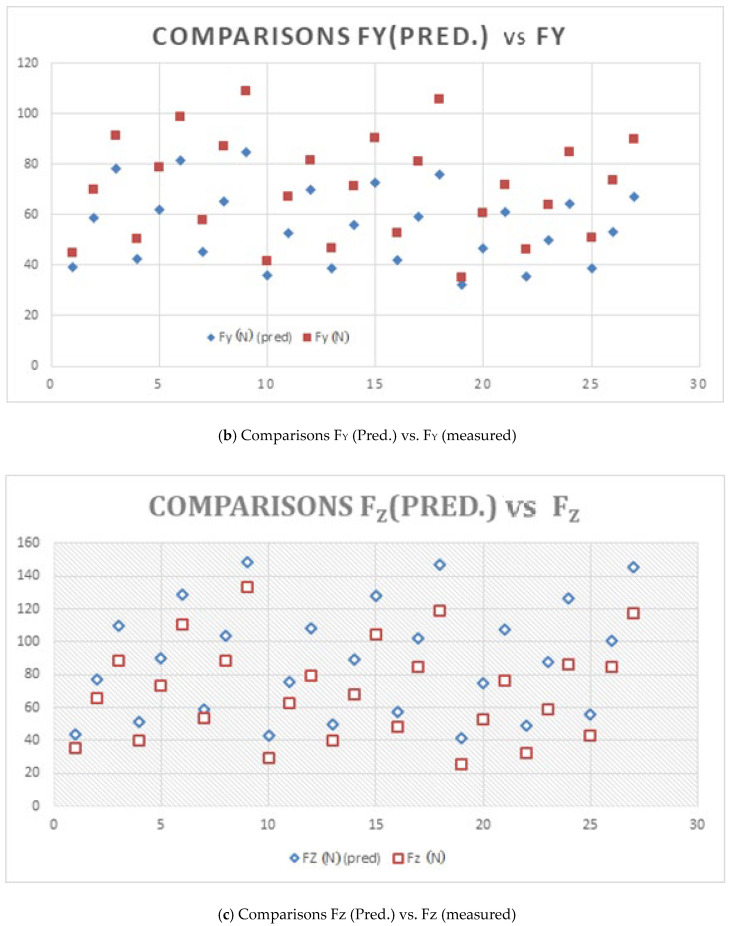
Comparisons of predicated and measured values.

The coefficient of the correlation R-sq. (adj) is 98.73%. We found that the predicted value of F_X_ was almost nearer to the measured value, which is shown in Figure 11a. Equation (2) shows a data accuracy of 98.73%.

The Fy model is given by:

(3) Fy=−12.001+0.139v+157.729f+107.21d−0.718v×d

The coefficient of the correlation R-sq. (adj) is 98.68%. We found that the predicted value of F_Y_ was almost nearer to the measured value, which is shown in Figure 11b with 98.68% accuracy.

The Fz model is given by:

(4) Fz=−9.47−0.08v−229.14f+84.07d+1188f×d 

The coefficient of the correlation R-sq. (adj) is 98.92%. We analysed the fact that the predicted value of F_Z_ was in full agreement with the measured value, which is shown in Figure 11c, with 98.92% accuracy.

## 4. Conclusions

In the current study, a turning operation was performed on an EN36B steel work-billet with a tungsten carbide tool. The machining variables, i.e., the cutting speed and feed and depth of cut, were optimised to reduce the wear rate. The concept of response-surface methodology was used for designing this experiment. ANOVA and linear regression analysis were performed to linearly fit the experimental data. It was concluded that the cutting-force components (Fx, Fy and Fz) are governed by the depth of cut. It was also found that flank-wear, i.e., the wear of the flank, generally increases linearly by the increasing cutting speed, depth of cut and feed rate. A mathematical model was established to show the difference between predicted flank-wear and the actual flank wear in the experiment; it was revealed that the predicted value of the flank-wear was almost nearer to the measured value, with a 96.33% to 98.92% confidence level.

In the future, this research work can be extended to optimize the values of surface roughness and material-removal rate (MRR), by varying the machining parameters. Artificial Neural Network and other optimization techniques can be used to investigate the thermal contact conductance and heat transfer phenomenon between cutting tools and material surface [49,50,51,52,53].

## Figures and Tables

**Table 1:** Cutting parameters and their level.

S. No.	Level	$\mathrm{Cutting}\;\mathrm{Speed}\;V\left(m/min\right)$	Feed Rate $f\;\left(mm/rev\right)$	Depth of Cut $d\;\left(mm\right)$
1 2 3	Low (−1) Medium (0) High (1)	40 55 70	0.04 0.06 0.08	0.50 0.75 1.00

**Table 2:** Chemical composition of EN36B.

S. NO.	Component	Min (%)	Max (%)
1 2 3 4 5 6 7	Carbon (C) Silicon(Si) Manganese (Mn) Phosphorus (P) Sulphur (S) Chromium (Cr) Nickel (Ni)	0.10 0.10 0.35 Nil Nil 0.70 3.0	0.160 0.0035 0.60 0.04 0.04 1.00 3.75

**Table 3:** Observation table obtained for cutting parameters speed, feed. and depth of cut.

S. No	v (m/min)	f (mm/rev)	d (mm)	F_X_ (N)	F_Y_ (N)	F_Z_ (N)	VB (mm)
1 2 3 4 5 6 7 8 9 10 11 12 13 14 15 16 17 18 19 20 21 22 23 24 25 26 27	40 40 40 40 40 40 40 40 40 55 55 55 55 55 55 55 55 55 70 70 70 70 70 70 70 70 70	0.04 0.04 0.04 0.06 0.06 0.06 0.08 0.08 0.08 0.04 0.04 0.04 0.06 0.06 0.06 0.08 0.08 0.08 0.04 0.04 0.04 0.06 0.06 0.06 0.08 0.08 0.08	0.5 0.75 1 0.5 0.75 1 0.5 0.75 1 0.5 0.75 1 0.5 0.75 1 0.5 0.75 1 0.5 0.75 1 0.5 0.75 1 0.5 0.75 1	15.125 33.375 65.36 16.355 42.82 69.05 17.515 45.06 71.315 14.67 33.135 53.13 16.345 37.32 61.335 17.065 38.065 66.3 13.84 32.025 50.73 14.62 33.165 58.545 16.63 35.135 58.545	44.835 69.82 91.395 50.225 78.77 98.865 57.98 87.22 108.85 41.605 67.235 81.385 46.61 71.32 90.375 52.48 80.96 105.745 35.285 60.46 71.575 46.15 63.63 84.685 50.885 73.79 89.81	35.335 65.42 88.165 40.2 73.21 110.84 53.555 88.485 133.17 29.295 62.46 79.3 39.805 68.09 104.685 48.01 84.805 119.05 25.29 52.925 76.225 32.37 58.53 86.355 42.685 84.325 117.08	0.321 0.373 0.433 0.34 0.414 0.453 0.359 0.412 0.452 0.346 0.403 0.459 0.366 0.418 0.479 0.381 0.438 0.498 0.372 0.425 0.482 0.37 0.444 0.504 0.385 0.49 0.548

**Table 4:** Result of analysis of variance (ANOVA) for VB, F_X_, F_Y_ and F_Z_.

Symbol	Degree of Freedom	Sum of Square	Mean Square	F	*p* Value	Contribution (%)	Remarks
**ANOVA for VB**							
**V** **f** **d** **v × f** **v × d** **f × d** **Error** **Total**	2 2 2 4 4 4 8 26	0.0119094 0.0067667 0.0635050 0.0006750 0.0008415 0.0002635 0.0009587 0.0849199	0.0059547 0.0033834 0.0317525 0.001688 0.0002104 0.000659 0.0001198	49.69 28.23 264.95 1.41 1.76 0.55	0.000 0.000 0.000 0.315 0.231 0.705	14.02 7.96 74.78 0.794 0.990 0.310 1.121	Significant Significant Significant Not Significant Not Significant Not Significant
**ANOVA for F_X_**							
**V** **f** **d** **v × f** **v × d** **f × d** **Error** **Total**	2 2 2 4 4 4 8 26	223.66 173.07 9463.08 5.09 102.14 36.98 39.11 10,043.14	111.83 86.54 4731.54 1.27 25.54 9.24 4.89	22.87 17.70 967.82 0.26 5.22 1.89	0.000 0.001 0.000 0.895 0.023 0.206	2.22 1.73 94.22 0.05 1.016 0.36 0.38	Significant Significant Significant Not Significant Significant Not Significant
**ANOVA for F_Y_**							
**V** **f** **d** **v × f** **v × d** **f × d** **Error** **Total**	2 2 2 4 4 4 8 26	695.36 1155.87 8801.33 11.38 92.68 44.70 44.08 10,845.4	347.68 577.94 4400.67 2.85 23.17 11.17 5.51	63.2 105.07 800.12 0.51 4.212 2.030	0.000 0.000 0.000 0.726 0.040 0.183	6.41 10.65 81.15 0.105 0.85 0.412 0.4057	Significant Significant Significant Not Significant Significant Not Significant
**ANOVA for F_Z_**							
**V** **f** **d** **v × f** **v × d** **f × d** **Error** **Total**	2 2 2 4 4 4 8 26	705.2 3723.3 17,948.3 67.1 62.3 474.7 76.6 23,057.5	352.59 1861.64 8974.14 16.78 15.58 118.68 9.575	36.82 194.42 937.2 1.75 1.627 12.39	0.000 0.000 0.000 0.231 0.258 0.002	3.058 1.616 77.84 0.291 0.2701 2.058	Significant Significant Significant Not Significant Not Significant Significant

## Data Availability

Not applicable.
